# The status of diagnosis and treatment to intracranial hypotension, including SIH

**DOI:** 10.1186/s10194-016-0708-8

**Published:** 2017-01-13

**Authors:** Jin-ping Lin, Shu-dong Zhang, Fei-fang He, Min-jun Liu, Xiao-xu Ma

**Affiliations:** 1 Department of Anesthesiology & Pain Management, Center for Intracranial Hypotension Management, Sir Run Run Shaw Hospital, School of Medicine, Zhejiang University, #3 East Qingchun Road, Hangzhou, 310016 Zhejiang Province People’s Republic of China; 2 Department of Gastroenterology, Sir Run Run Shaw Hospital, School of Medicine, Zhejiang University, #3 East Qingchun Road, Hangzhou, 310016 Zhejiang Province People’s Republic of China; 3 Department of Anesthesiology, Sir Run Run Shaw Hospital, School of Medicine, Zhejiang University, #3 East Qingchun Road, Hangzhou, 310016 Zhejiang Province People’s Republic of China

**Keywords:** Intracranial hypotension, Imaging tests, Epidural blood patching

## Abstract

Intracranial hypotension, especially spontaneous intracranial hypotension (SIH), is a well—recognized entity associated with cerebrospinal fluid (CSF) leaks, and has being recognized better in resent years, while still woefully inadequate. An increasing number of factors including iatrogenic factors are realized to involve in development and progression of intracranial hypotension. The diagnosis remains difficult due to the various clinical manifestations, some of which are nonspecific and easily to be neglected. Multiple imaging tests are optional in CSF leakage identification while clinicians are still confronted with difficulties when making selection resulting from superiorities and disadvantages of different imaging tests. Treatments for intracranial hypotension are multifarious but evidence is anecdotal. Values of autologous epidural blood patching (EBP), the mainstay of first-line interventional treatment currently, is getting more and more regards while there are no systematic review of its efficacy and risks. Hereby, the purpose of this review was to reveal the present strategy of intracranial hypotension diagnosis and treatment by reviewing literatures, coupled with our experience in clinical work.

## Review

### Cerebrospinal fluid physiology

Approximately 500 ml of cerebrospinal fluid (CSF) is produced daily, with a volume of about 150 ml replaced three to four times everyday through the choroid plexus system for cushion the brain and spinal cord [1]. As measured using manometry in lateral position via lumbar puncture, CSF opening pressure in adults is 70–200 mm H_2_O related with body mass index (BMI), the larger BMI is, the higher CSF pressure is [2–4]. In children, larger pressure variance and higher opening pressure ranges were demonstrated [5] while no normative data on the elder’s CSF pressure was found.

### Epidemiology

Intracranial hypotension, including spontaneous intracranial hypotension (SIH) which was firstly described by Schaltenbrand in 1938, arises and clinical symptoms appear when the CSF pressure beyond the auto-regulation. The associated prevalence and incidence has been estimated at 2–5:100,000, [6–10], with the higher in large scale comprehensive hospital and the lower in local health-center. Female are affected twice to fifth as often as males [8, 11]. The peak incidence is in 30–50 year-old although it also occurs in children and the aged [8, 11–16].

### Etiology

It is generally known that CSF leakage is the most common feature of intracranial hypotension, and substantial majority of cases had cervical or thoracic localization of CSF leakage [11, 13] while the specific etiology of underlying spontaneous CSF leaks remains largely undetermined. For sure, various dural weakness, either congenital or contrived, ranging from simple dural tears to multi-level complex meningeal diverticula, allows CSF to leak into the epidural [17, 18]. Above all, patients with symptomatic intracranial hypotension with a history of dural puncture should be first consideration [19]. Minor trauma, mainly refer to tumble, is reported in 80% patients [20]. Connective tissue disorders such as Marfan syndrome, polycystic kidney disease, Ehlers-Danlos syndrome Type II, neurofibromatosis and Lehman syndrome are also involved in spinal CSF leakages which all associated with genetic heterogeneity [11, 21–24]. As we mentioned above, female are affected twice to fifth as often as males, and this suggests female hormone mediated effects on dural integrity [8, 11]. Attention should be paid on vertebral osteophytic spur which can result in dural puncture reported by Williams [25]. Additionally, other relevant factors include malnutrition [26], short stature [27] and etc. Some specialists demonstrated that CSF leakage at the skull base may not associated with orthostatic headaches [13, 28], and this hypothesis was approved clearly by Schievink in 2012 [13]. Until now, no significant data suggested that SIH is caused by decreased CSF secretion or generalized CSF hyperabsorption [28].

### Clinic features

Intracranial hypotension is characterized by diffuse pachymeningeal enhancement on cranial MRI features, low CSF pressure and orthostatic headaches mostly caused by the dural puncture entitled postdural puncture headache (PDPH) [1, 29, 30] with a 4 to 50% incidence calculated by researchers [31–33]. Orthostatic headache is defined as development or aggravation of headache when patients move from a supine to an upright position for seconds or minutes and headache disappears or typically relieves after lay down for a while [1, 34–36]. According to Monro–Kellie hypothesis, the CSF leakage reduces the CSF pressure and may give rises to a venous dilatation which is considered to cause headache via meningeal traction and subdural effusions, hematomas via the rupture of bridging veins [37, 38]. Dramatically, the orthostatic headache is not diagnostic and features of it sometimes are variable. It might be gradual or thunderclap in onset [8, 39], less or more positional over time, even intermittent [40], no relationship with position [41] or become worse when lying down [42], and the intensity of headache was variable [12] or absence of headache [12, 43]. Lagrand [44] reported a severe SIH case of orthostatic unconsciousness might caused by diencephalic herniation [45, 46]. Other cardinal symptoms reported are nausea and vomiting occurred in about half of patients [8, 47], always accompanied by neck pain stiffness [26], blurred vision [12], “visual” filed defects and diplopia [48], cough headache [30], facial pain or numbness [49], tinnitus [41], taste alternations [8, 47] and limb paresthesias [50] and transient third cranial nerve palsy [51]. Neurocognitive decline such as dementia [52, 53], behavioral changes [54–56] and parkinsonism [57] also can be presented which is rarer but has also been report.

### Diagnose and image features

The diagnosis of intracranial hypotension is based on the combination of medical history, clinical features and imaging findings [58]. However, due to the woefully inadequate awareness of it, the ratio of initial misdiagnosis and missed diagnosis is pretty high, and it also make contribute to the long mean time (13 months) from symptom onset to diagnosis [47], eventually the headache disorder can evolve into a chronic daily headache pattern [59]. In order to improve the terrible situation of diagnosis and treatment and perfect guide significance, The International Headache Society developed the 3^rd^ diagnostic criteria (Table 1) [60] for SIH in 2013 on the basis of the criteria developed previously [61] which was too restrictive for clinic works [34, 62]. Compared with the 2^nd^ criteria allowing the diagnosis to be made with the absence of abnormal imaging results [34] and the one proposed by Mea [62] et al., the 3^rd^ criteria requires documentation of low CSF pressure and an association of the low pressure with the headache, and there should be a CSF pressure of <60 mm H_2_O or evidence of CSF leakage on imaging. Exclusion of other disorders is necessary such as postpartum, venous sinus thrombosis and subdural hematoma which are well known to cause positional headache [63].

**Table 1:** International Classification of Headache Disorders (ICHD) diagnostic criteria for SIH [60]

A. Any headache fulfilling Criterion B
B. Headache has developed in temporal relation to low CSF pressure or CSF leakage, or has led to its discovery
C. Low CSF pressure (<6 cm H_2_O) and/OR evidence of CSF leakage on imaging
D. Not better accounted for by another ICHD-3 Diagnosis

Intracranial hypotension is a well-recognized entity associated with CSF leakage [54]. Multiple imaging methods are highly applicative and valuable for suspicious CSF leakage diagnosis [34], while some matters are needed.

#### Cranial CT

Bilateral subdural hematomas, effacement of the subarachnoid cisterns and ventricular collapse are shown as CSF leakage features by brain CT. However, despite brain CT does not provide as many cardinal features as MRI, it is prior to more definitive imaging in the outpatients or emergency department setting and it is a helpful initial diagnostic tool for its non-invasive.

#### Cranial MRI

Generally, there are some nonspecific characteristic imaging features most often associated with SIH. Subdural fluid collection which is the most common feature [8, 26, 64], pachymemningeal enhancement, engorgement of venous structures, pituitary hyperemia and sagging of the brain relevant with effacement of perichiasmatic cisterns, bowing of the optic chiasm, flattening of the pituitary stalk and pons, effacement of the prepontine cisterns, descent of the cerebellar tonsils within the posterior fossa and so on. And yet, 20–30% of patients with SIH may have a normal brain MRI [12, 34, 58, 65].

#### Spinal MRI

Efficiency of spinal MRI in localizing CSF leaks is lower than CT myelography (CTM), while some specific spinal manifestations of CSF leaks can be demonstrated in it such as dilated epidural and intradural veins, dural enhancement and meningeal diverticulae [66, 67]. Although plain spinal MRI may yield false-negative results because a collapse of the dural sac and epidural CSF accumulations are easily missed, it is still an excellent method of demonstrating the extent of ventral spinal CSF leaks.

#### Myelography

CTM and intrathecal gadolinium magnetic resonance myelography (Gd-MRM) both have higher sensitivity and better spatial resolution to localize spinal CSF leaks when compared with radionuclide cisternography. Standard CTM is best to localize suspected slow leaks and dynamic CTM is best to localize suspected fast leaks, which would otherwise spread over many levels, obscuring the actual leakage site [25, 68]. Generally, Gd-MRM has higher sensitivity than CTM for small, slow leaks, and is the optimum selection for spinal meningeal diverticula and dural ectasia in patients no leak was identified on a prior CTM, but it is more complex to perform [69]. Despite increased sensitivity, CTM and Gd-MRM fail to identify a CSF leak in close to 30% of subjects with suspicious SIH [68, 69]. So for contrast-enhanced Gd-MRM, the intrathecal injection of contrast medium is straightforward and safe which can be performed in the case of suspicious findings on unenhanced images [70–72].

#### Radionuclide Cisternography

When intracranial hypotension is suspicious, the use of radionuclide cisternography does have some value. The main features of it are slow ascentalong the spinal axis and a relative lack of activity over the convexities. Radionuclide Cisternography has been largely superseded by CTM and is increasingly considered obsolete due to its poor resolution and non-invasive quality of CTM [8, 70, 72, 73]. Its limited usefulness as the site of CSF leak remains unknown in up to one third of patients [74].

Spinal CSF leakage can be identified in most, but not all, intracranial hypotension cases. In those without a demonstrable leakage by imaging tests, it has been postulated that the spinal CSF leak is intermittent or below the level of detection of current imaging techniques [13], so the time when to take imaging tests is in significance.

### Treatment

Intracranial hypotension often recovered spontaneously and no further treatments are needed [75, 76]. Although evidence is anecdotal [32, 77, 78], conservative medical management should be processed when SIH is highly suspected while necessary auxiliary examinations proceeding and many patients respond to it alone [8, 12]. For mild intracranial hypotension patients, conservative approaches include strict bed rest, appropriate liquid, caffeine and theophylline, analgesic, non-steroidal drugs and abdominal binders [25, 59, 77–79]. Using steroids remains controversial, which might be beneficial for regulating negative feedback of nervous and immune system. For PDPH, vasodilator agent therapy (mixture of 5%CO_2_ and 95%O_2_ with flow 5–10 ml/h) is optional and very effective by dilating vessel, lower vascular obstruction and increase CSF secretion while other conservative measures are less efficient for it [80].

When initial conservative approaches fail to bring relief of clinical symptoms and for moderate and severe patients, antilogous epidural blood patching (EBP) should be considered, it is current the mainstay of first-line interventional treatment and universally effective, especially there are explicit leakage [12, 34, 79, 81–84]. EBP is the optimum method for PDPH and also can be applied reasonably in SIH [25, 85]. Repeated EBPs should be offered according to at least one-fourth of patients are not cured by one blood patch and about 50% cases require more than one blood patch [81, 82]. Repeated EBPs are efficacious in many refractory SIH [86]. Obviously, the management of patients whose symptoms persist despite two or more EBPs performed becomes increasingly difficult and complex [35, 54, 84].

Surgery should be considered for patients whose leak sites are clearly identified and no adequate response to all non-surgical measures has been observed and two or more EBPs had been performed [6, 15, 34, 87]. Surgeries include ligation of leaking meningeal diverticula, direct repair of dural tears, packing of the epidural space with fibrin glue or strengthening the dura with a duroplasty [88]. Seriously, these surgical methods are insufficient of evidence and should be carried out by specialists with empiric in managing refractory SIH.

There is approximate 0.5–1% of all headache presentations to Emergency Department [10]. In keeping with the case Lagrand [44] reported, very few patients characterized by alterations in conscious levels leading to coma or even death, which caused by severe sagging brain, subdural hematoma or venous sinus thrombosis. In emergency, adequate amount of intrathecal saline infusion can be a useful temporizing measure to normalize the intracranial pressure and so gain time to investigate and correct the CSF leak [89], as Stephen described in an instructive case report [90]. In addition, same with other surgical measures, the surgical evacuation of subdural hematomas is still in dispute, and only can be provided after the leakage of cerebrospinal fluid was handled.

### Information of EBP

SIH was firstly described by Schaltenbrand in 1938 and EBP was firstly introduced for treatment of PDPH in 1960 by Gormley. EBP means injecting antilogous venous blood into epidural space within minutes in prone position under aseptic conditions. EBP acts as a mass lesion compressing the dura and raising intracranial pressure by increasing CSF volume [35, 91–93], the blood clot which formed to seals off the dural hole [94–96] and finally the CSF leak stops. Hereby, this might be the reason saline used in epidural injection is no longer currently because it fails to close the hole in the dura causing persistent CSF volume loss [84, 97]. In clinic, when the location of CSF leakage is unclear, *blind EBP* is recommended from lumbar avoiding performing it through an in situ epidural catheter as lumbar puncture [35]. Controversially, in Szeinfeld’s opinion, a same injection site of EBP with the lumbar puncture site is advisable [98]. Alternatively, if the CSF leakage identified, *target EBP* should be provided under CT or fluoroscopic guidance with a 50 to 100% symptomatic improvement rate [64, 82, 99–102]. The success rate is best in these receiving a target blood patch at the symptom onset, but this need to be balanced against the higher potential of complications [59]. For instance, due to the anatomy, the epidural space becoming smaller from the lumbar ascend to the cervical region, it is technically more challenging for clinician to perform epidural punctures in the upper thoracic and cervical spine [54].

Still, the volume of blood optimizes the efficacy of an EBP remains uncertain [76]. Inconsistently, the recommended volumes for EBP which has no business with the height of the patients [31] range from 10 ml up to 20 ml after clinicians attempted various volume of blood ranged over 2 to 50 ml [9, 54, 98, 103–106], and the largest volume was 130 ml performed by Graff [26]. A higher success rate was reported using nearly 20 ml [106, 107]. Griauzde [54] and Ohtonari [108] recommends using a large-volume blood patch in multiple CSF leak sites and refractory cases through a single-catheter access site for the treatment of SIH, and this is partially contrary with Taivain’s opinion that a larger volume EBP in adults does not produce a better effect on PDPH with a standard 10 ml volume [31]. Based on the above comprehensive situation, it is particularly necessary to point out that the EBP procedure should be discontinued when the patient complain pain in the back or legs [98] regardless of the volume of injected blood.

However, there are no controlled studies about of antilogous EBP in CSF leaks yet [21], utilization of it has just been based on the clinical observation of its effectiveness [34]. It is considered initially successful when headache disappeared completely instantaneous, always during the 2 h recovery room follow-up, and the success rate was about 84.5–97.5%, while the overall success rate with a permanent cure was only 61% which might resulted from psychological factors [9, 31, 104]. In our institute, significant clinical improvement was obtained in 183 SIH patients after targeted EBP was performed once (88%), twice (7%) or thrice (5%) on the first admission, without the long-term follow-up achieved.

Some factors are involved in the success rate of EBP. For instances, using the Trendelenburg position before, during and after the EBP procedure has been reported to improve the success rate in over half of cases [12, 65] because this position might reduce the flow of spinal CSF fistula [12] and anti-Trendelenburg position increased the flow of CSF leaks [109]. Inadequate blood volumes, early patching, steroids in the epidural space and dural punctures performed with large bore needles were postulated to be possible causes of EBP failure [27, 110]. Another contested issue is utility of target EBP. Ferrante [12] claimed that providing EBP from lumbar as effective as CT-directed EBP even the leaks are identified, this opinion is in conflict with majority clinicians’ proposition [31, 35]. It is well acknowledged that acetazolamide might avoid the rebound intracranial hypertension sometimes reported after the EBP [111], in Ferrante’s research whether the use of acetazolamide improved the success rate is unknown, further investigation are needed.

Agree with other authors’ advise [12, 82], we also recommend days of conservative treatments before using EBP, perform EBP precociously few days after the beginning of symptoms [59]. Avoiding lumbar puncture in typical cases because such a procedure aggravates CSF seep and normal CSF pressure does not exclude the diagnosis of intracranial hypotension. In severe case, even with complications as coma and second lumbar EBP failed, invasive test should be rejected as much as possible and a target EBP is more effective than the lumbar one [12, 112].

It would be specially mentioned that some complications of EBP exit though they are rare and usually not long-lasting. They are inclusive of cranial nerve palsies, intracranial hypertension, paraparesis, cauda equina syndrome, aseptic arachnoiditis, chemical or infectious meningitis, subdural and subarachnoid hematoma, pneumocephalus, and so on [31, 96, 113–115]. Repeated injections of blood into the epidural space may potentially lead to fibrosis and obliteration of the epidural space [35, 104, 105], so it is vitally important for clinicians to balance the advantages and drawbacks of repeat EBPs.

### Prevention of iatrogenic risk factors

Risk factors for intracranial hypotension are variable and some are avoidable. Despite the nonmodifiable risk factors such as age, female gender, lower BMI, history of prior PDPH and chronic headache, modifiable risk factors play crucial roles in the occurrence and prognosis of orthostatic headache, hence, imperative concern should be paid on it such as shape, size of spinal needle, bevel orientation and angle of insertion, and even the operator experience.

Adequate needle shape like Atraumatic needle including Sprotte and Whitacre reduces the incidence of PDPH because dural holes created by it are more likely to close and return to their original position after the needle withdrawn and minimizing the damage to the dura [116–121]. In order to overcome the disadvantage of Atraumatic needles such as deeper insertion, dull tip makes it more difficult to penetrate the skin [19], the use of an 18-gauge introducer together with the spinal needle can help circumvent the difficulty with skin penetration [94].

Gilland [2] demonstrated that statistically significant were observed in CSF pressure using 22-gauge needle and 26-gauge needle, the smaller gauge the needle is, the lower CSF pressure is. The best estimate of the true CSF pressure should be the one obtained with a 26-gauge needle, which is an often overlooked principle in clinic practice. However, in young cases, a high incidence of PDPH has been reported even use 26-gauge needle dew to the dural hole exists created by it and the pressure gradient [122–124]. Geurts [91] and Tourtellott [124] revealed that the incidence of PDPH and backache were much higher using 22-gauge and 25-gauge needles than 29-gauge ones, and there were no PDPH had been observed when using the latter, and sizes of needles are non of business with respect to obtaining adequate spinal anaesthesia and spread of blockade. However, the smaller needle is, much more difficult the procedure is. Moreover, PDPH is less common if the bevel of a traumatic needle of any design is oriented and removed parallel to the long axis of the spine rather than perpendicular to it to reduced injury for branching elastic fibers resulting in a smaller dural opening [33, 125–127]. All above information on needles are instructive for clinic work according to different purposes.

Although bed rest, hydration, the volume of CSF removed during dural puncture, times of attempts and pretreatment with oral and intramuscular caffeine have not been shown to be associated with reduced PDPH incidence [32, 77, 128, 129], prophylaxis with intravenous caffeine resulted in significantly lower pain scores, less severe headaches, and decreased analgesic use compared to placebo [130]. Using frovatriptan and acetazolamide pretreatment might be protective [12, 131]. It is equally important that lower incidence of inadvertent dural puncture during epidural anesthesia was observed among experienced clinicians [96, 132]. In short, the skills of clinicians are vital in intracranial hypotension diagnosis and treatment, especially in invasive measures.

## Conclusions

Intracranial hypotension, especially SIH, is much more common than generally realized and poses considerable diagnostic and treatment challenges. There are still so many controversies on its treatment, so innovative therapies should be investigated because some patients are refractory to any treatment. The most urgent thing is that our clinicians should improve the awareness and skills in intracranial hypotension diagnosis and treatment.
